# Evaluation of current algorithms for segmentation of scar tissue from late Gadolinium enhancement cardiovascular magnetic resonance of the left atrium: an open-access grand challenge

**DOI:** 10.1186/1532-429X-15-105

**Published:** 2013-12-20

**Authors:** Rashed Karim, R James Housden, Mayuragoban Balasubramaniam, Zhong Chen, Daniel Perry, Ayesha Uddin, Yosra Al-Beyatti, Ebrahim Palkhi, Prince Acheampong, Samantha Obom, Anja Hennemuth, YingLi Lu, Wenjia Bai, Wenzhe Shi, Yi Gao, Heinz-Otto Peitgen, Perry Radau, Reza Razavi, Allen Tannenbaum, Daniel Rueckert, Josh Cates, Tobias Schaeffter, Dana Peters, Rob MacLeod, Kawal Rhode

**Affiliations:** 1 Department of Imaging Sciences & Biomedical Engineering, King’s College London, London, UK; 2 Utah Center for Advanced Imaging Research, University of Utah, Salt Lake City, Utah, USA; 3 Magnetic Resonance Research Centre, Yale School of Medicine, Yale University, New Haven, USA; 4 Department of Computing, Imperial College London, London, UK; 5 School of Electrical and Computer Engineering, Boston University, Boston, USA; 6 Psychiatry Neuroimaging Lab, Harvard Medical School, Boston, USA; 7 Imaging Research, Sunnybrook Health Sciences Centre, Toronto, Canada; 8 Fraunhofer Institute for Medical Image Computing, Fraunhofer MEVIS, Bremen, Germany; 9 Beth Israel Deaconess Medical Center, Harvard Medical School, Boston, USA

**Keywords:** Late gadolinium enhancement, Cardiovascular magnetic resonance, Atrial fibrillation, Segmentation, Algorithm benchmarking

## Abstract

**Background:**

Late Gadolinium enhancement (LGE) cardiovascular magnetic resonance (CMR) imaging can be used to visualise regions of fibrosis and scarring in the left atrium (LA) myocardium. This can be important for treatment stratification of patients with atrial fibrillation (AF) and for assessment of treatment after radio frequency catheter ablation (RFCA). In this paper we present a standardised evaluation benchmarking framework for algorithms segmenting fibrosis and scar from LGE CMR images. The algorithms reported are the response to an open challenge that was put to the medical imaging community through an ISBI (IEEE International Symposium on Biomedical Imaging) workshop.

**Methods:**

The image database consisted of 60 multicenter, multivendor LGE CMR image datasets from patients with AF, with 30 images taken before and 30 after RFCA for the treatment of AF. A reference standard for scar and fibrosis was established by merging manual segmentations from three observers. Furthermore, scar was also quantified using 2, 3 and 4 standard deviations (SD) and full-width-at-half-maximum (FWHM) methods. Seven institutions responded to the challenge: Imperial College (IC), Mevis Fraunhofer (MV), Sunnybrook Health Sciences (SY), Harvard/Boston University (HB), Yale School of Medicine (YL), King’s College London (KCL) and Utah CARMA (UTA, UTB). There were 8 different algorithms evaluated in this study.

**Results:**

Some algorithms were able to perform significantly better than SD and FWHM methods in both pre- and post-ablation imaging. Segmentation in pre-ablation images was challenging and good correlation with the reference standard was found in post-ablation images. Overlap scores (out of 100) with the reference standard were as follows: Pre: IC = 37, MV = 22, SY = 17, YL = 48, KCL = 30, UTA = 42, UTB = 45; Post: IC = 76, MV = 85, SY = 73, HB = 76, YL = 84, KCL = 78, UTA = 78, UTB = 72.

**Conclusions:**

The study concludes that currently no algorithm is deemed clearly better than others. There is scope for further algorithmic developments in LA fibrosis and scar quantification from LGE CMR images. Benchmarking of future scar segmentation algorithms is thus important. The proposed benchmarking framework is made available as open-source and new participants can evaluate their algorithms via a web-based interface.

## Background

In the past decade, there has been a rapid development of analysis tools in medical imaging. In contrast, their translation to the clinical environment has remained limited. A major contributing factor for this failure is lack of proper validation strategies. Even though algorithms are tested in-house extensively following development, it is often not clear how they perform relative to other *state-of-the-art* algorithms. The main reason for this is they are not compared using the same set of data. Differences in evaluated datasets (i.e. patient type, image quality and resolution) makes a fair comparison difficult.

Benchmarking of algorithms is thus a very important activity as we move from bench to bedside in the medical image processing community. In the last few years, several conferences in the medical image analysis field have provided a platform to benchmark algorithms from multiple research groups. These *challenges* have been organised to invite participants to test their algorithms on common data. The participants are given a number of training datasets and then asked to complete analysis of a number of unseen data within an allotted time. Following submission, the algorithms’ results are evaluated in a unified manner.

In the past few years, a number of collaborating research groups have set up a publicly available evaluation frameworks for the medical image processing and analysis community. Most of them have been initiated through an organised challenge and an index of past challenges can be found in http://www.grand-challenge.org/. In the cardiac imaging domain, some recent challenges include cardiac motion tracking [1] and coronary artery stenosis detection [2].

### Motivation for left atrial fibrosis/scar segmentation challenge

There is a great interest in understanding the mechanisms of the causes of atrial fibrillation (AF) and of pulmonary vein (PV) reconnection following ablation procedures [3]. Late Gadolinium enhancement (LGE) cardiovascular magnetic resonance (CMR) imaging plays an important role in the management of AF. Recent work has demonstrated its use in assessment of atrial fibrosis before ablation and of atrial injury after ablation [4-8].

Segmentation of fibrosis or scar in LGE CMR is challenging due to multiple causes including the thin LA wall, contrast variation due to inversion time, signal-to-noise ratio, motion blurring and artefacts [8]. The inversion time choice can generate the appearance of more or less scar, and change the appropriate scar threshold. Motion blurring also reduces the appearance of scar. There are also artefacts which appear in the image due to respiratory compensation, selectively reducing the ability to visualise scar in the right PVs. There is also the complex geometry of the LA, resulting in some transverse slices where a very small section of the anatomy is visible, particularly for left and right superior PVs. There are also many regularly enhancing structures, such as the aortic wall, the valves and the oesophagus, which must be distinguished from LA enhancement.

As CMR plays an increasingly important role in the quantification of pre-ablation fibrosis and post-ablation scar, development of reliable algorithms that remove observer bias is key for clinically useful quantification. To our knowledge, there is no standardised evaluation framework or methodology to evaluate the performance of existing or newly developed LGE CMR segmentation.

### State-of-the-art for cardiac fibrosis/scar segmentation

Here we give an overview of the previously published fibrosis or scar detection, quantification and segmentation algorithms and report on how they were evaluated. Refer to Table 1 for a brief summary. A common method for detecting fibrosis or scar is the application of a threshold two or three standard deviations above the average intensity value of a healthy myocardial region [9-11]. Others such as the full-width-at-half-maximum (FWHM) can be used [12] and some use thresholding to further classify scar into core or peri-core regions [13].

**Table 1:** Overview of previously published scar detection, quantification and segmentation methods

**Reference**	**Model**	***n***	**Modality**	**LV/LA**	**Algorithm**	**Evaluation**
Kim et al. [9]	Canine	26	CMR	LV	SD	Infarct size, *ex-vivo*
Amado et al. [12]	Animal	13	CMR	LV	SD, FWHM	Bland altman, Infarct volume
Kolipaka et al. [10]	Human	23	CMR	LV	SD	Percentage scar, Bland-Altman
Positano et al. [14]	Human	15	CMR	LV	Clustering	Percentage scar
Yan et al. [13]	Human	144	CMR	LV	SD	Percentage scar
Schmidt et al. [11]	Human	47	CMR	LV	SD	Infarct size
Hennemuth et al. [15]	Human	21	CMR	LV	EM fitting	Percentage scar, Bland-Altman
Oakes et al. [5]	Human	81	CMR	LA	SD	Percentage scar
Detsky et al. [16]	Human	15	CMR	LV	Clustering	Infarct size
Tao et al. [17]	Human	20	CMR	LV	Otsu thresholding	Dice
Knowles et al. [4]	Human	7	CMR	LA	MIP	Percentage scar
Lu et al. [18]	Human	10	CMR	LV	Graph-cuts	Infarct size and Bland-Altman

Methods were analysed on the type of data they were evaluated with and the structure of interest: left ventricle (LV) or left atrium (LA). The number of datasets (*n*) is listed. Most methods employed simple standard deviation (SD) thresholding from a base healthy tissue intensity value. Others such as full-width-at-half-maximum (FWHM), maximum intensity projection (MIP) and expectation-maximisation (EM) fitting have also been proposed. The evaluation measures used were compared.

Other approaches exist to compute the threshold automatically [10] or apply clustering [14,16], or with Graph-cuts [18]. Visualization of infarcted regions with maximum intensity projections (MIP) is also possible [4] which is useful for visualising the amount of scarring on the LA surface. For detection of pre-ablation fibrosis, a global threshold for the image can be computed and adjusting it on a slice-by-slice basis provides good detection [5].

All of the existing methods reviewed except for [5] and [4] detect scar in the ventricle myocardium. Segmenting scar in the atrium poses different challenges especially from nearby enhancing structures such as aortic wall and valves. The atrial myocardium is of smaller thickness compared to ventricular myocardium and this adds to the difficulty of the problem. It is also important to understand that using a fixed model (SD and FWHM) is not suitable for the atrium and in our opinion also for the ventricle despite several studies utilising this. The reasons are clear: a fixed model cannot handle all the different variabilities encountered and these are both from the varied internal (size, distribution and heterogeneity of scar) and varied external (resolution, image noise, inversion time, surface coil intensity variation) situations. And there is at least one study supporting this fact - in [5] where it was shown that the threshold had to be re-adjusted on various slices to obtain a suitable segmentation.

### Proposed evaluation framework

In this paper we present an evaluation framework, accessible via a web-based interface, for algorithms that segment LA fibrosis or scar from both pre- and post-ablation LGE CMR images. The presented results were submitted as a response to the open challenge that was put to the medical imaging community through the cDEMRIS (Cardiac Delayed Enhancement Segmentation Challenge) workshop organised as part of the ISBI 2012 (IEEE International Symposium on Biomedical Imaging) annual meeting. Each participant quantified the amount of fibrosis or scar in high-resolution 3D LGE CMR of 30 pre- and 30 post-ablation patients. There were in total 7 institutions who responded to the challenge, and segmentation results from 8 different algorithms were submitted. The datasets used in this evaluation are publicly available via the challenge website: http://www.isd.kcl.ac.uk/cdemris/.

The proposed evaluation framework aims to provide a platform for testing and comparing newly devised algorithms through a web-based interface. With 3 out of the 8 algorithms evaluated in this work already published in literature [5,15,18], the framework provides a valuable test-bed.

## Methods

### Data acquisition database

**Figure 1:**
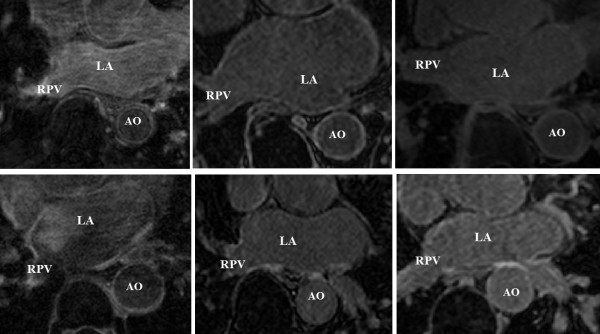
**Challenge LGE CMR data sample.** A sample of the CMR data included in the challenge. The pre-procedural (top-row) and post-procedural (bottom-row) LGE images are shown. Abbreviations: AO - aorta, LA - left atrium, RPV - right pulmonary vein.

LGE CMR images of the LA of varying quality, resolution and parameters were selected from three imaging centres. These centres were Utah School of Medicine, Beth Israel Deaconess Medical Center (BIDMC) and Imaging Sciences at King’s College London (KCL-IM) (see Table 2). Images were acquired either pre- or post-ablation. A total of 60 images were collected. These were 30 images taken at pre- and 30 images at post-ablation. Each centre provided 10 images each of pre- and post-ablation. The time of acquisition of pre-scans varied slightly between 1 to 7 days depending on the imaging centre. For post scans this was more variable with either 1 month or between 3 to 6 months (See Table 2). A wide spectrum of images were selected to get a representative range from typical clinical acquisitions in the datasets. Images of variable quality were chosen, especially in relation to enhancement quality. The collected database also included segmentation of the LA endocardium and cavity for each LGE CMR scan. This was also provided as part of the challenge and it was optional for the participant to utilise it. Representative images are shown in Figure 1.

**Table 2:** Image acquisition: image acquisition parameters for the challenge LGE data

	**U. Utah**	**BIDMC**	**KCL-IM**
**Scanner type**	Siemens Avanto 1.5T or Vario 3T	Philips Acheiva 1.5T	Philips Achieva 1.5 T
**Basic params**	Free-breathing (FB) with navigator-gating	FB and navigator-gating with fat suppression	FB with navigator-gating with fat suppression
**TI **^**†**^**, TR, TE**	300 ms, 5.4 ms, 2.3 ms	280 ms, 5.3 ms, 2.1 ms	280 ms, 5.3 ms, 2.1 ms
**Acquired resolution**	1.25 × 1.25 × 2.5mm	1.4 × 1.4 × 1.4mm	1.3 × 1.3 × 4.0mm
**Pre-scan**	< 7 days	< 7 days	< 48 hours
**Post-scan**	3 - 6 months	= 30 days	3 - 6 months

†- set to null myocardium.

*Abbreviations*: *TI* Inversion time, *TR* Repetition time, *TE* Echo time. Imaging centres: *U. Utah* University of Utah, *BIDMC* Beth Israel Deaconess Medical Center, *KCL-IM* Imaging Sciences, King’s College London.

A brief summary of the algorithms evaluated for this framework is given in Table 3. They are described in greater detail in the section below with a very brief background on the technique implemented and details of the implementation.

**Table 3:** A brief summary of algorithms that were evaluated on the proposed framework

**Algorithm**	**Technique**	**Evaluation**	**Atrial wall**	**Strengths**	**Weaknesses**
**IC**: Bai et al.	Hysteresis thresholding	30 pre and post	Euclidean distance - 3 mm	Coherent segmentations	Fixed sigmoid models derived from empirical data
**MV**: Hennemuth et al.	Region-growing with EM-fitting	30 pre and post	Euclidean distance - 3 mm	Post ablation imaging	Pre-ablation imaging
**SY**: Lu et al.	MRF model with graph-cuts	20 pre and post	Dilation - 4 mm	Fuzzy membership - improved delineation	Post-processing for small cluster removal
**HB**: Gao et al.	Active contour and EM-fitting	15 post	Active contour (snake)	Accurate myocardial segmentation	Fixed number of gaussian mixtures in model (i.e. two)
**YL**: Peters et al.	Simple thresholding	15 pre and post	Manual	Accurate segmentation on both pre- and post.	Time consuming
**KCL**: Karim et al.	MRF model with graph-cuts	30 pre and post	Post-ablation imaging	Pre-ablation imaging	Post-processing steps necessary
**UTA**: Cates et al.	Histogram analysis and simple thresholding	30 pre and post	Manual	Accurate segmentation on pre and post.	Time consuming
**UTB**: Perry et al.	*k*-means clustering	30 pre and post	Manual	Pre-ablation fibrosis	Equivalent variance across all clusters - LA scar variance more variable

Institution *abbreviations*: *IC* Imperial College, *MV* Mevis Fraunhofer, *SY* Sunnybrook Toronto, *HB* Harvard/Boston University, *YL* Yale School of Medicine, *KCL* King’s College London, *UTA/B* Utah School of Medicine.

### Algorithm 1: Imperial college - hysterisis thresholding (IC)

#### Background

Hysteresis thresholding was used in this work to segment scar. It is a well-known approach in image processing and computer vision [19]. It is an improvement over regular thresholding where a major drawback is the absence of coherence in the final segmentation. Hysteresis thresholding overcomes this because faint sections of atrial scar can also be segmented as long as they are adjacent to some salient sections.

#### Implementation

To model enhancement in scar pixels, pixel intensities *I*(*x*) were first normalized according to:

(1) $$\overset{ˆ}{I}(x)=\frac{I(x)-\mu_{B}}{\sigma_{B}}$$

where *μ*_*B*_,*σ*_*B*_ are mean and standard deviation of LA blood pool cavity respectively. Based on the normalized intensity value, the enhancement was modelled with a sigmoid function. The model outputs a probability *p*_*i*_(*x*) based on the normalised intensity:

(2) $$p_{i}(x)=\frac{1}{1+e^{-(\overset{ˆ}{I}-c_{i})/h_{i}}}$$

where *c*_*i*_ and *h*_*i*_ are parameters of the sigmoid function. As scar should only be located in atrial myocardium, the likelihood of scar decreases with increasing distance from LA endocardium, and this was modelled with:

(3) $$p_{d}(x)=\frac{1}{1+e^{-(d(x)-c_{d})/h_{d}}}$$

where *c*_*d*_ and *h*_*d*_ are parameters of the sigmoid function and *d*(*x*) is the Euclidean distance from LA endocardium. The joint probability of both the intensity and distance likelihoods, i.e. *p*(*x*) = *p*_*d*_(*x*) · *p*_*i*_(*x*) was used to generate a probabilistic map. Using hysteresis thresholding, pixels above the higher threshold limit were classified as foreground. Those above the lower threshold limit and connected to foreground were also classified as foreground. This was accomplished by exploring a foreground pixel’s neighbourhood and thus this ensured coherence in the segmented result.

### Algorithm 2: Mevis - Region growing with mixture model fitting (MV)

#### Background

Region growing is an important segmentation technique for finding groups of connected pixels with intensity homogeneity. It was implemented in this work with thresholds selected both for region-growing and seed selection using Gaussian mixture models.

#### Implementation

For scar segmentation, good seed locations are those within regions that are highly likely to be scar. In this submission, to obtain good seed voxels, a Gaussian mixture model with three mixtures was used to model three separate intensity levels: LGE, atrial wall and blood (*B*) and neighbouring structures (*N*):

(4) $$h(x)=\underset{i\in \{\text{LGE},B,N\}}{\sum }\alpha_{i}\frac{1}{\sqrt{2\pi \sigma_{i}}}e^{\frac{1}{2}\left[\frac{x-\mu_{i}}{\sigma_{i}}\right]}$$

where *h*(*x*) is the mixture model with three weighted (*α*_*i*_) mixtures in *LGE*,*B* and *N* each with a mean *μ*_*i*_ and standard deviation *σ*_*i*_. The mixture was fitted to the LGE CMR intensity distribution of the LA. Seed selection was performed by using a lower intensity threshold cut-off at *I*_*s*_:

(5) $$I_{s}>0.15\cdot \mu_{B}+0.85\cdot \mu_{\text{LGE}}$$

where *μ*_*B*_ and *μ*_*LGE*_ were obtained from the fitted mixture model *h*(*x*) in Eq. 4. Following seed selection, region growing was initiated from each seed with an intensity threshold *I*_*R*_ as:

(6) $$I_{R}>\min\{\mu_{\text{LGE}},I_{t}\}$$

where *I*_*t*_ is the intensity at the intersection of blood and LGE mixtures: *B* and *LGE*. It is expected that at this intersection, LGE intensities starts contributing more than blood intensities. Region growing was constrained within a 6 mm band around the endocardial segmentation allowing 1 mm inside and 5 mm outside the endocardial surface. This allowed for any errors in the endocardial contour.

### Algorithm 3: Sunnybrook - Graph-cuts with fuzzy c-means clustering (SY)

#### Background

The proposed technique uses graph-cuts and a modified version of this algorithm is published in [18]. In mathematics, a *graph* is a network of nodes connected by links. Each link can be assigned a weight. An image contains pixels, each of which can be represented with a node. Adjacent pixels or nodes can then be interconnected with links. This allows an image to be modelled as a graph. Numerous problems have been proposed and solved on graphs, for example shortest path through two nodes or partitioning the graph into two node sets.

For the task of binary image segmentation, pixels are grouped or partitioned into two disjoint sets. Similarly, graph-cuts is an approach of partitioning a graph into two or more sub-graphs with some imposed constraints. Two special nodes called *source* and *sink* nodes are assigned, with each node in the graph linked to them. These nodes represent labels of the segmentation (i.e. foreground and background). Each link to the source and sink is weighed based on the probability of the node for the label. A minimum cut through the graph can then be computed, partitioning it into two sets of nodes. Each set is connected to source or sink. This essentially computes a segmentation of each pixel into a label. The minimum cut and maximum flow are dual problems both investigated thoroughly in mathematics [20,21] and computer vision [22,23].

#### Implementation

The method of graph-cuts is applied in this work to segment scar in LGE CMR images. Starting from the provided LA endocardial segmentation, the atrial wall myocardium was approximated by dilating the endocardial boundary by 4 mm. A graph of interconnected neighbouring pixels was constructed for all pixels within the computed atrial wall myocardium. Links were also created to the source and sink nodes representing scar and healthy tissues. Each pixel ended up having two types of links: 1) links to source and sink, 2) links to its adjacent pixels. A weight or energy was assigned to each link. The two weights are summarised in this energy formulation *E*(*L*):

(7) $$E(L)=\lambda \underset{x}{\sum }R_{x}\left(L_{x}\right)+(1-\lambda )\underset{(x,y)\in N}{\sum }B_{\text{xy}}\left(L_{x},L_{y}\right)$$

where *L* = {*L*_*x*_|*x* ∈ *X*} denotes a segmentation of all pixels *X*. *N* is the set of adjacent pixel pairs. *R*_*x*_ is the weight for links to source/sink nodes and *B*_*xy*_ is the weight for links between adjacent pixels. The *λ* term weighs the influence of these terms in the energy function. In this work, *R*_*x*_ was obtained by computing a c-means fuzzy clustering [24] on the computed atrial myocardium region. Following clustering, each pixel attained a fuzzy membership which directly contributed to *R*_*x*_(*L*_*x*_). *B*_*xy*_ was obtained using a function that penalised intensity dissimilarity between adjacent pixels:

(8) $$B_{\text{xy}}(L_{x},L_{y})=\frac{e^{-\beta {\left|I_{x}-I_{y}\right|}^{2}}}{d(x,y)}$$

where *d*(*x*,*y*) is Euclidean distance between pixels *x* and *y* and *β* is a penalty co-efficient fixed at 5 in this work. This value was chosen to increase the relative importance of high gradient between pixels of different classes, refer to [18] for further details.

### Algorithm 4: Harvard/Boston University - Active contours and mixture model fitting (HB)

#### Background

Two techniques are implemented in this work, namely active contour and the Expectation-Maximization (EM) algorithm. A brief background is given here on each technique. Further details can be found in [25].

Active contours [26] was used in this technique to obtain the epicardial boundary. It counteracts the issue of region leaking in region growing. This is possible by imposing constraints on the growing region. An initial contour was modelled with a spline (i.e. a free-form curve) allowing it to grow flexibly with additional constraints placed by the image. An energy function captured these constraints and the final shape of the contour was obtained through energy minimisation.

The expectation-maximization (EM) algorithm [27] is a technique for estimating model parameters given the observed data. The observed data in this submission are the distributions of atrial wall image intensities and the model is a statistical Gaussian mixture model. The EM algorithm computes the best estimate of model parameters for which the observed data are most likely. It alternates between the E-step which computes the expectation of the likelihood of observed data using a present estimate of model parameters and the M-step that re-computes model parameters by maximising the likelihood found in the E-step.

#### Implementation

The left atrial wall can be challenging to segment in LGE CMR especially due to two reasons: 1) thickness, and 2) lack of enhancement making wall difficult to detect. In this work, prior to segmenting scar, atrial wall is obtained by segmenting the epicardium. As the LA endocardium is made available as part of the challenge data, a simple subtraction of epicardium to endocardium obtains the wall. Active contours are used to accomplish the epicardium segmentation task. In 3D, active contours can be extended into surfaces. Let us denote such a deformable surface *S* and an energy function *E*(*S*) constraining its deformation:

(9) $$E(S)=\int_{S}\left((1-\lambda )f(x)+\lambda {\left(d(x)-3\right)}^{2}\right)\text{dx}$$

where *d*(*x*) is the Euclidean distance function and 3 mm is the expected size of the atrial wall; *f*(*x*) represents a simple function of the image intensity gradient:

(10) $$f(x)=\frac{1}{1+G_{\sigma }*\nabla I(x)}$$

where the intensity gradients ∇*I*(*x*) are smoothed using a Gaussian filter *G*_*σ*_. This evolves the deformable surface governed by *E*(*S*) and restricts it with a combination of distance from endocardium (i.e. maximum 3 mm) and intensity gradient. The evolution must stop at the epicardial border where an intensity change is expected.

Following segmentation of atrial wall, scar is classified from healthy tissue by modelling the distribution of intensities within atrial wall as a mixture of two Gaussians. The Gaussians mixture represent scar and healthy tissue. The mean and standard deviation of each Gaussian in the mixture model is determined using the EM-algorithm.

### Algorithm 5: Yale - Threshold selection with manual wall delineation (YL)

#### Background

Simple thresholding is a fundamental technique in image segmentation. Thresholding is used in this work to segment scar from both pre-ablation and post-ablation images. The main disadvantage of thresholding is that only intensity information is considered and the relationships between pixels is not taken into account. Thus, there is no guarantee that the pixels identified by thresholding are contiguous.

#### Implementation

**Figure 2:**
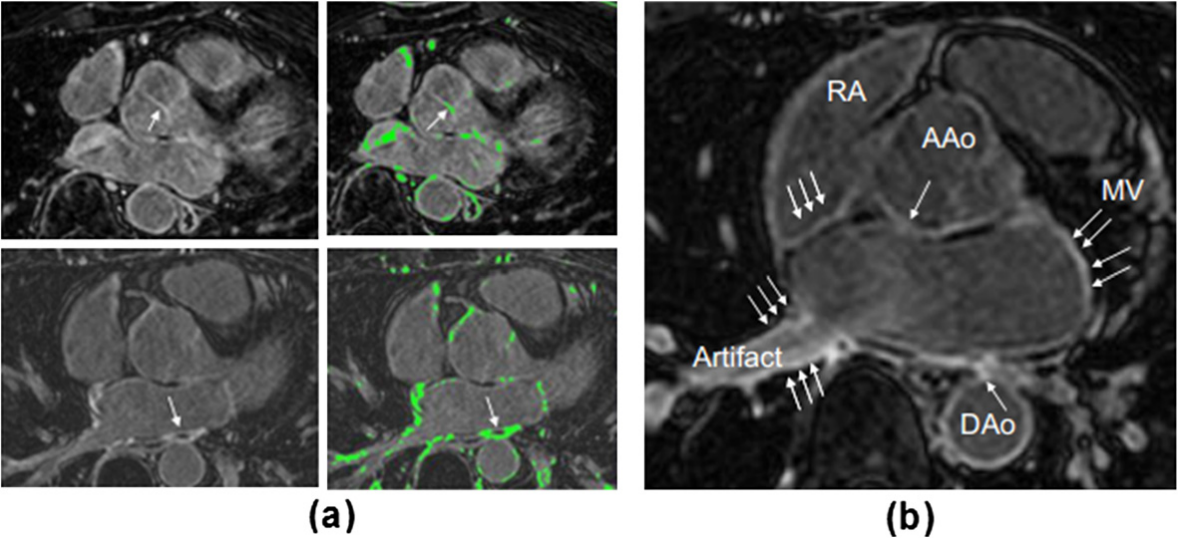
**Yale method’s threshold criterion.** **(a)** Choice of threshold for pre-ablation (top) and post-ablation (bottom) images. Arrow points to fibrosis used for choosing threshold. Pre-ablation, this was a prominent section of the aortic valve. Post-ablation, the entire area of a prominent scar was selected. **(b)** The LA wall identification excludes regions where enhancement existed, but was attributed to artefacts or fibrosis of the mitral valve (MV), Aortic wall (AAo, DAo), or right atrial (RA) wall (arrows).

There are two important considerations in this work: 1) threshold selection for fibrosis and scar, and 2) manual delineation of the regions of the atrial wall myocardium which will be subject to this thresholding. The criteria for selecting threshold are different for pre- and post-ablation images. For pre-ablation images, the average intensity of the enhancement around the mitral valve was used (see Figure 2(a)). This is reasonable since valves are known to be fibrotic and usually visible in LGE CMR images. For post ablation images, the threshold was set to include an entire region of prominent scar (as shown in Figure 2(a)). A single threshold is used for the entire 3D volume. The criteria for including atrial wall for further thresholding are described in Figure 2(b), and include avoidance of the mitral valve and aortic wall enhancement and artifactual enhancement.

### Algorithm 6: KCL - Graph-cuts with EM-algorithm (KCL)

#### Background

A background of the techniques used in this work is described above in Sections 'Algorithm 3: Sunnybrook - Graph-cuts with fuzzy c-means clustering (SY)’ (Graph-cuts) and 'Algorithm 4: Harvard/Boston University - Active contours and mixture model fitting (HB)’ (EM-algorithm). More details can be found in [28].

#### Implementation

Scar was segmented both in pre- and post-ablation images using the graph-cut algorithm [22]. A statistical distribution model of scar tissue in both pre- and post-ablation images was developed prior to segmentation. This distribution model was derived from a training set of images. As a training set was not provided as part of the challenge, the leave-one-out approach was used for training with 29/30 images for training and 1/30 for testing. The training distribution model is a Gaussian distribution of the scar intensities in the training image represented as a ratio of scar to average blood-pool. Scar was segmented manually by an experienced observer.

The intensity distribution model for non-scar or healthy tissue was obtained from the target or unseen image. A Gaussian mixture was used for this distribution model. The number of mixtures in the model was kept variable (1 to 5) depending on the configuration which best fits the image. The standard EM-algorithm computed mean and variance for each mixture. Only a region 3 mm inside and outside the LA endocardium was used for the EM fit, discarding the rest of the image. This also became the search space for scar.

Pixels within the search space were modelled as a graph network with paths to source and sink nodes (i.e. scar and healthy tissue labels). The path to the scar tissue label was assigned a probability value from the scar training distribution model and the path to the healthy tissue label was assigned a probability value from the non-scar distribution model. Paths between adjacent pixels were assigned a probability value based on intensity homogeneity, with a low probability value for dissimilar intensities. All of the above is captured with an energy function which is the standard graph-cut functional and is equivalent to Eq. 7.

### Algorithm 7: Utah A - Threshold selection with manual wall delineation (UTA)

#### Background

The method was primarily implemented for pre-ablation fibrosis. However, in this challenge, its results on post-ablation data was also submitted. Thresholding is used in this work and is described above in Section 'Background’. The method is also described in detail in [5].

#### Implementation

The atrial wall myocardium is delineated prior to scar segmentation. An experienced observer delineated the wall in every slice. Using the intensity histogram of pixels within the delineated wall, a threshold for scar was calculated. It is expected that the histogram is bi-modal with modes for enhancement and non-enhancement intensities. The threshold was then computed as +2-4 standard deviations off the mean of the lower mode of the histogram. This threshold was adjusted for every slice based on whether the algorithm was over- or under-estimating scar.

### Algorithm 8: Utah B - Unsupervised learning using *k*-means clustering (UTB)

#### Background

The method uses *k*-means clustering which is a machine learning approach used to identify the optimal number of pixel groups or clusters [29]. It is an unsupervised learning technique requiring no prior knowledge or training data. In *k*-means clustering, the number of possible clusters is specified. It is an iterative process, where at each iteration the centre of each cluster is updated and membership of each point to a cluster is updated based on a pre-defined distance/error metric in the feature space.

#### Implementation

The technique was primarily implemented for post-ablation scar. However, in this challenge, its results on pre-ablation data was also submitted. There were two important considerations for the implementation of *k*-means: 1) the number of clusters in the *k*-means algorithm and 2) the feature vector for comparing pixels. Prior to segmentation, the optimal number of clusters and feature vectors were determined through empirical evaluation. The number of clusters was varied between 3 to 10 and image features such as normalised voxel intensity, the Sobel filter and the 14 texture metrics proposed by Haralick et al. [30] were tested. The optimal number of clusters was found to be 4 with normalised voxel intensity as the feature vector. Following *k*-means clustering, the cluster with the highest mean intensity was assigned as the scar cluster.

### Algorithm evaluation

#### Reference standard 1: pseudo-ground truth

In order to obtain a reference standard for scar, volumetric segmentations of scars were obtained from three separate observers. These observers have substantial experience looking at scars in LGE CMR images for both pre- and post-ablation images. The observers were from different centres. They were blinded to the image scanner manufacturers and also to the results of the challenge. Scars in the images were segmented as follows: 1) each axial slice in the LGE CMR image was analyzed separately. Segmentation of the LA endocardial body was loaded as an overlay; 2) pixels enhanced along the endocardial border were labelled as scar; and 3) segmentations were also corrected in coronal and sagittal slices, wherever necessary.

Although the observers were provided with the same guidelines, their segmentations differed in some instances especially in images with low contrast enhancement ratio. It was thus important to merge the segmentations and obtain a consensus. This was possible by merging segmentations using the STAPLE algorithm described in [31]. For each voxel, a probability estimate for the true segmentation was computed. The consensus segmentation can then be obtained by thresholding this probability above 0.7 or 70%. This is referred in the rest of the text as the pseudo-ground truth.

#### Reference standard 2: *n*-SD and FWHM

The optimal method for quantifying scar from LGE CMR images yet remains unclear. However, certain methods have been adopted for obtaining scar using a fixed model. In these fixed models, signal intensity of normal myocardium is measured and a certain number of SD from this measured intensity is used as the threshold. Although in [32] this threshold was set to 2-SD, recently it was shown that FWHM was far more reproducible and reliable than 2-SD [33]. Other cut-offs are also used: 3,4,5 or 6-SDs. The FWHM technique, which uses half the maximal signal within a hyper-enhanced region in scar, is currently being advocated as the most reproducible technique for ventricle myocardial scar [33].

In order to gauge each challenger’s methodology against fixed-model quantification methods, the LGE CMR images were segmented using 2, 4, 6-SD and FWHM methods. For each method, a segmentation of atrial myocardium was necessary and this was approximated by dilating the endocardial wall 3 mm. For the *n*-SD methods, an expert observer located a region of voxels in atrial myocardial that was healthy. The mean and SD of this region were calculated. Voxels with intensity greater than 2, 4, 6-SD, in the atrial myocardium, were labelled as scar. For the FWHM method, an experienced observer identified an enhanced region within atrial myocardium. The threshold was then set to 50% of the maximum intensity in this selected region. In some rare instances, the 50% cut-off was adjusted to 60% or 70% when a 50% cut-off was too low for the image.

#### Evaluation metrics

To evaluate the performance of each challenger’s segmentations, they were compared against the pseudo ground-truth. Since there is no single metric which works best for evaluating segmentations, a few different metrics were chosen for evaluating them. These were regional, volumetric and surface-based metrics. This allowed us to effectively test the reproducibility and accuracy of each method. Segmentations from *n*-SD and FWHM were also compared using the same metrics. This allowed each challenger’s algorithm to be gauged against these published techniques. We briefly describe each evaluation metric: 

1. Regional metric: The Dice similarity co-efficient was used as a regional metric. It measures the proportion of true positives in the segmentation:

(11) $$s=\frac{2|X\cap Y|}{\left|X|+|Y\right|}$$

where *X* is the region in ground-truth and *Y* is the region in the challenger’s algorithm. The Dice was measured both on the entire image and also locally. Since Dice is a regional metric comparing single voxels, when measured on images as a whole, the Dice only gives the algorithm’s average performance. An equal weighting is given to every slice, even though some slices may only have a few pixels in the segmentation. An algorithm may do very well in slices that matter and yet be penalised for slices that have a small number of enhancing voxels. To counteract this issue, the Dice was computed for selected local regions within each image. An experienced observer selected several regions within each image where: 1) there was enhancement and the consensus segmentation agreed or, 2) there was enhancement but consensus segmentation did not agree (i.e. artefacts). The Dice was computed individually for these regions.

2. Surface-based metric: It is common to visualise segmentations of scar on the LA surface. This is usually possible with a MIP. The LA surface can be constructed as an iso-surface from a volumetric binary segmentation using the marching cubes algorithm [34]. Scar segmentation is MIP-ed and each surface mesh vertex attains a label (1 = scar,0 = not scar). The surface-based metric measures the root-mean-squared-error (RMSE) between vertex points labelled as scar in the algorithm’s output and ground-truth distance. The RMSE is given by:

(12) $$\text{RMSE}=\sqrt{\frac{1}{N}\overset{N}{\underset{i=1}{\sum }}d{\left(g_{i},t_{i}\right)}^{2}}$$

where {*g*_*i*_ : *i* = 1,…,*N*} is the set of mesh vertex points labelled as scar in the ground truth and {*t*_*i*_ : *i* = 1,…,*N*} labelled as scar in the test or algorithm output image. Also, *d* is the Euclidean distance function.

3. Volumetric-based metric: The total volume error between the challenger’s segmentation and pseudo ground truth was found:

(13) $$\delta V=|V_{T}-V_{G}|$$

where *V*_*T*_ is the volume of scar in the segmentation and *V*_*G*_ is the volume of scar in consensus segmentation.

#### Objective evaluation

Acquisition artifacts and non-scar related enhancement are common in atrial LGE CMR scans. Unless these enhancements are explicitly modelled into the technique, it is challenging to distinguish them. Two sources of non-scar related enhancements commonly seen in atrial LGE CMR images are: 1) the navigator beam artifact often seen near the right PVs, and 2) Gadolinium uptake by the aortic wall and valves. To test whether the methods are able to handle un-related enhancements, each challenger’s segmentations were evaluated separately in these regions. An experienced observer selected regions containing navigator artefacts and aortic wall enhancements. The percentage of voxels detected by each method in these spurious regions was determined. This gave an indication of the proportion of false positives.

A good contrast between normal myocardium, blood pool and scar is desirable and is the most technically challenging part of LGE CMR image acquisition. The quality of contrast depends on achieving the optimal inversion time. Each post-ablation image was scored by three raters experienced in LGE CMR images and the average score was taken. Images in the database (only post-ablation scans) were ranked into three categories: good, average and poor. The Dice metric was computed separately in each category. This indicated how robust the algorithms were against contrast enhancement quality.

## Results

In this section results from our evaluation are presented with figures and plots.

### Segmentation accuracy with pseudo ground truth

For each LGE CMR scan available for the challenge, a pseudo ground truth was available by combining manual segmentations of scar from three experienced observers as described in Section 'Reference standard 1: pseudo-ground truth’.

**Figure 3:**
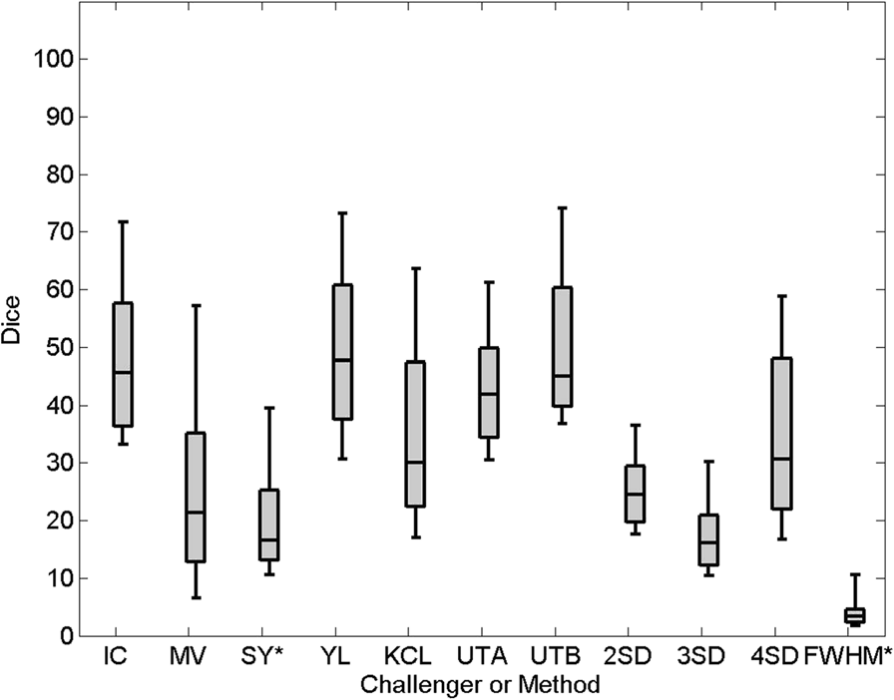
**Performance on pre-ablation LGE CMR images.** Dice overlap scores in selected regions on pre LGE CMR scans. An asterix(*) denotes challengers who did not submit segmentations on all patients. Note that the figure also displays results from the 2-SD, 3-SD, 4-SD and FWHM methods.

**Figure 4:**
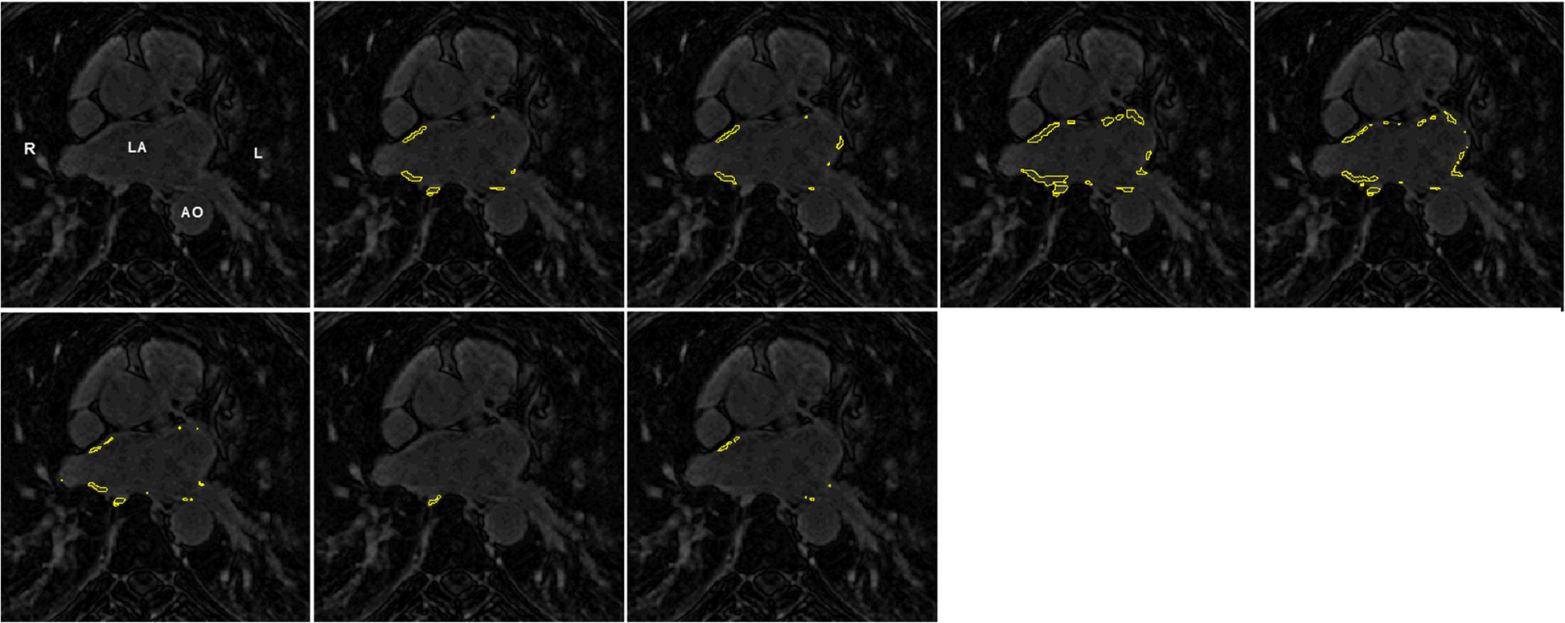
**Sample segmentations from pre-ablation data.** Segmentations from a pre scan. Clockwise from top-left: original LGE CMR scan, consensus segmentation, IC, MV, SY, UTB, UTA, KCL, YL. Abbreviations: L- left side, R- right side, LA - left atrium, AO - aorta.

On the pre-LGE CMR scans, segmentation accuracies of each challenger were compared. However, accuracy could not be computed for challenger HB as they provided no segmentations on the pre-data. Figure 3 shows the Dice overlap scores for all participants on pre-LGE CMR scans. The median Dice overlap shown in the plot are as follows: IC = 37, MV = 22, SY = 17, YL = 48, KCL = 30, UTA = 42, UTB = 45. Published methods for segmenting scar such as 4-SD and FWHM were also tested on the pre-data and the Dice overlap scores for these were: 2-SD = 24, 3-SD = 16, 4-SD = 31 and FWHM = 5. Examples of segmentations from a single slice are seen in Figure 4.

**Figure 5:**
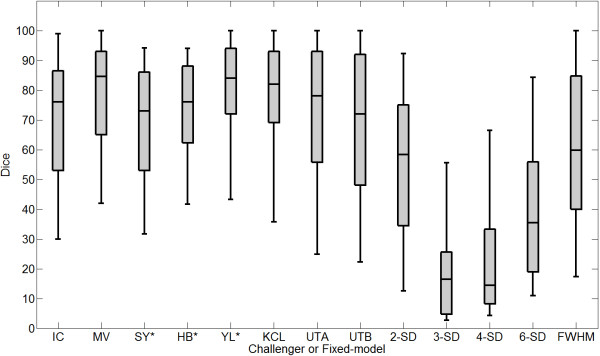
**Performance on post-ablation LGE CMR images.** Dice overlap scores on post LGE CMR scans. An asterix(*) denotes challengers who did not submit segmentations on all patients. Note that the figure also displays results from the 2-SD, 3-SD, 4-SD, 6-SD and FWHM methods.

**Figure 6:**
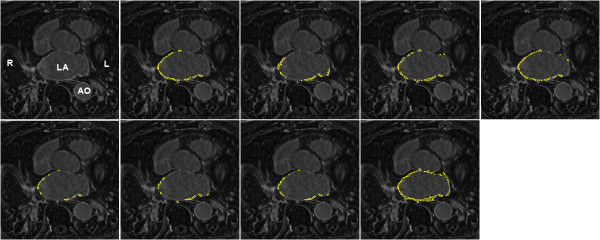
**Sample segmentations from post-ablation data.** Segmentations from a post scan. Clockwise from top-left: original LGE CMR scan, consensus segmentation, IC, MV, SY, UTA, UTB, KCL, YL, HB. Abbreviations: L- left side, R- right side, LA - left atrium, AO - aorta.

On the post-LGE CMR scans, segmentation accuracy of each challenger was evaluated in a similar way to the pre-data. Figure 5 shows Dice overlap scores of all participants on post-LGE scans. The median Dice overlap shown in the plot are as follows: IC = 76,MV = 85,SY = 73,HB = 76,YL = 84,KCL = 78,UTA = 78,UTB = 72. However, note that some participants (SY, HB and YL) did not submit segmentations on all scans and their Dice overlap scores are on a smaller cohort of scans compared to other challengers who submitted segmentations on all thirty scans. Examples of segmentations from a single slice are seen in Figure 6.

Methods using a fixed-model, such as *n*-SD and FWHM for segmenting scar in LGE CMR images, were tested on the post-data. Figure 5 shows Dice overlap scores on post- LGE scans using *n*-SD and FWHM. The median Dice overlap were found to be: 2-SD = 58,3-SD = 17,4-SD = 14,6-SD = 35,FWHM = 59. Apart from using the Dice overlap for measuring accuracy, the RMSE and volume difference were also computed. Table 4 lists the RMSE and volume differences in pre- and post- data for all algorithms. However, there are some exceptions. As HB provided no submission on the pre-data, the metrics for these could not be computed. In addition, SY and YL provided 20 and 15 (out of total 30) for both pre- and post- data respectively.

**Table 4:** **Segmentation accuracy with root-mean-squared-error (RMSE) and volume difference (*δ*** ***V*) on pre and post data for both submitted algorithms (IC to UTB) and fixed-models**

	**Pre data**		**Post data**	
	**RMSE (mm)**	**|** ***δV*** **| (ml)**	**RMSE (mm)**	**|** ***δV*** **| (ml)**
IC	0.72 (0.5)	2.87 (2.0)	9.52 (8.2)	4.79 (2.9)
MV	1.42 (0.7)	38.08 (6.7)	9.20 (8.8)	4.15 (5.7)
SY ^†∗^	0.17 (0.1)	12.87 (2.8)	9.22 (9.3)	10.19 (3.9)
HB ^∗^	n.a.	n.a.	n.a.	20.16 (10.3)
YL ^†∗^	1.03 (0.4)	0.62 (0.7)	6.34 (8.2)	2.77 (2.3)
KCL	1.33 (0.6)	2.24 (2.2)	9.20 (8.3)	3.10 (2.3)
UTA	0.36 (0.3)	3.24 (2.6)	10.72 (8.0)	3.54 (2.5)
UTB	0.52 (0.5)	3.10 (2.2)	8.91 (8.2)	1.25 (1.5)
2-SD	n.a.	7.51 (3.6)	n.a.	17.7 (10.1)
3-SD	n.a.	12.73 (8.3)	n.a.	7.64 (3.7)
4-SD	0.15 (0.1)	12.74 (8.3)	11.69 (7.5)	11.98 (8.5)
6-SD	n.a.	n.a.	n.a.	15.47 (8.5)
FWHM	n.a.	70.52 (38.4)	7.67 (8.2)	6.61 (5.9)

The standard deviation of each metric is quoted in brackets. Symbols (†∗) for pre and (∗) for post denote algorithms that could only be tested on a subset of the complete set of images. *Abbreviations*: *n.a.* data not available or could not be computed.

### Non-scar enhancing structures

**Figure 7:**
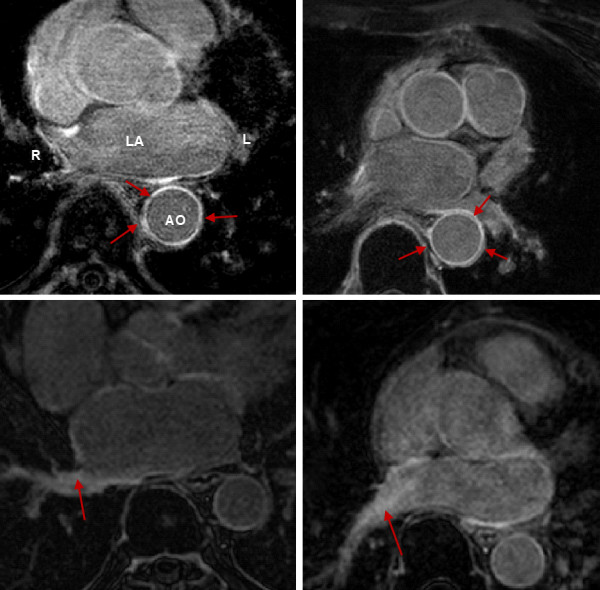
**Non-scar enhancing structures in LGE CMR images.** Images show examples of regularly enhancing structures (first row) and enhancement due to the navigator beam (second row). Arrows indicate enhanced sections of interest. Abbreviations: LA - left atrium, Ao - aorta, L - left, R - right.

**Figure 8:**
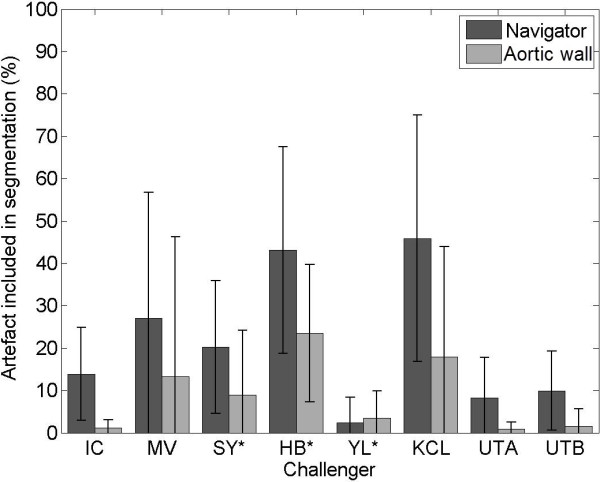
**Artefact analysis.** Amount of artefact (navigator beam artefact in the right superior pulmonary vein and enhancement in aortic wall) included in segmentations of each challenger. An asterix(*) denotes challengers which could not be assessed on all artefact samples.

There are various regularly enhancing structures in LGE CMR images, for example the aortic wall or valves that should be differentiated from scar. Some examples are shown in Figure 7. For both pre- and post-LGE CMR scans of the challenge, the amount of enhancements not related to scar detected by each method was quantified. They were compared against enhancements separately labelled by an experienced observer and deemed to be highly unlikely from scar. These labels were divided into two categories: aortic wall enhancement and navigator beam artefact. The total volume detected by each method was represented as a percentage of the total volume labelled by the observer. The results are represented in Figure 8. KCL and HB detected on average between 40-50% of total non-scar enhancements labelled by the observer. This value for IC, MV, SY, YL, UTA and UTB was between 5%–30%, with YL less than 5%.

### Image quality on segmentation

The LGE CMR images included in this challenge were acquired at three imaging centres with differing protocols and scanners (see Table 2). The quality of enhancement is known to vary and this variation across the imaging centres was quantified. Further the LGE CMR images were qualitatively classified based on their quality and the algorithms evaluated accordingly.

**Figure 9:**
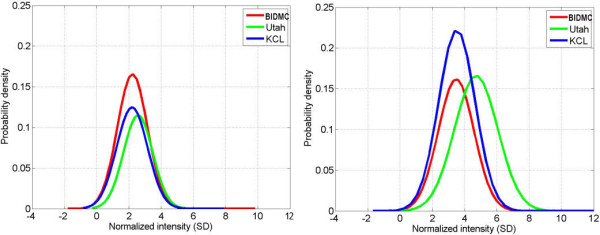
**Enhancement quality at imaging centres.** Variation in enhancement quality: Enhancement normalisation in images (*n* = 60) from all three centres supplied for the challenge in pre-ablation (left) and post-ablation (right) LGE CMR. The histograms plot enhanced pixels in the consensus segmentation. Intensities are normalised to atrial blood pool mean. Horizontal axes represents intensity in enhancement as standard deviations (SD) from blood-pool mean. Increasing enhancement corresponds to increasing SD.

To quantify quality of enhancement, using images from all three centres, histograms of signal intensity of enhanced regions in the pseudo ground truth, presented as SDs above the mean blood pool signal were computed. These histograms can be seen in Figure 9 and was separately quantified for each imaging centre. In both pre- and post-ablation images, the quality of enhancement did not vary greatly, except for Utah in post-ablation: pre-ablation (BIDMC, Utah, KCL-IM) = (2.2 ± 0.9, 2.5 ± 0.9, 2.1 ± 0.9), and post-ablation: (BIDMC, Utah, KCL-IM) = (3.5 ± 1.1, 4.7 ± 1.3, 3.5 ± 1.2). These values provided the basis for selecting 2-SD, 3-SD, 4-SD and 6-SD cut-offs in the fixed models used for establishing the reference standard. However, even with selecting optimal cut-offs: 2- to 3-SD for pre-ablation and 3- to 4-SD for post-ablation images, results from Section 'Segmentation accuracy with pseudo ground truth’ suggest that these settings may not yield the best segmentations. This can be explained by the amount of variation in enhancement quality of images from a particular centre: 36-42% for pre-ablation and 27-32% for post-ablation images. Thus a fixed model was found to suffer for these reasons.

The LGE CMR images were qualitatively classified based on enhancement quality and classified into three categories: good, average and poor. A good scan had both reasonably good signal-to-noise ratio and contrast ratio for enhanced areas. The algorithms’ accuracy were evaluated based on image quality, the Dice metric was computed separately for post scans in each category. Results are given in Table 5. No significant drop in performance was found with any of the methods (WilCoxon rank-sum test). For most algorithms, a marginally higher Dice is seen on better quality scans but this was not significant. The fixed-model methods (2-SD to FWHM) performed predictably with slight reduction in accuracy going from good to poor quality scans.

**Table 5:** Analysis of segmentation accuracy based on image quality (good, average and poor) on post-scans

**Challengers**	**Good**	**Average**	**Poor**
	**Mean (SD)**	**Mean (SD)**	**Mean (SD)**
IC	64 (26)	64 (27)	69 (23)
MV	83 (20)	80 (21)	79 (20)
SY*	70 (21)	64 (26)	71 (22)
HB*	76 (17)	71 (21)	74 (16)
YL*	80 (20)	73 (25)	74 (24)
KCL	78 (18)	77 (25)	73 (26)
UTA	64 (29)	70 (29)	71 (28)
UTB	63 (28)	67 (28)	67 (24)
2-SD	56 (27)	53 (29)	53 (27)
4-SD	17 (19)	21 (27)	17 (15)
6-SD	38 (21)	35 (25)	34 (20)
FWHM	68 (30)	66 (27)	56 (34)

The mean and standard deviation (SD) of the Dice metric is given for each challenger (IC to UTB) and fixed-model methods (2-SD to FWHM).

## Discussion

We presented a standardised evaluation framework, accessible via a web-based interface, that allows the effective comparison of scar segmentation algorithms in the LA for pre- and post-ablation fibrosis and scar. The framework has been used to compare eight algorithms as part of the cDEMRIS challenge, a workshop organised at ISBI in 2012. The data is publicly available via the website: http://www.isd.kcl.ac.uk/cdemris/.

### Evaluation framework

The usefulness and effectiveness of an evaluation framework is important. The evaluation framework presented in this work comprised thirty pre-ablation and thirty post-ablation image database from three separate imaging centres (KCL-IM, Utah and BIDMC) acquired using scanners of two different vendors (Siemens Healthcare and Philips Healthcare). Further, images differed in slice-thickness (1.25 - 2.0 mm reconstructed) and acquisition time-point (1-7 days for pre- and 30 - 180 days for post-ablation). This ensured that algorithms would not be biased towards a specific acquisition protocol. The selection of images for the framework was not random. They were carefully chosen to include images that exhibited artefacts (navigator, aortic wall, valve fibrosis), poor contrast-noise ratio and poor enhancement. Thus the presented framework provides a wide spectrum of data suitable for testing algorithms.

Two reference standards are established within the framework: the algorithms were tested against consensus segmentations of multiple observers and established techniques *n*-SD and FWHM. The task of creating a reference standard from multiple observers is complex and tedious. The observers were provided with set guidelines. Although, their delineations were approximately consistent, some differences remained. It was thus important to merge the segmentations with STAPLE [31]. For instance in images with poor contrast enhancement ratio, observers may differ in their opinion of the level of enhancement that is likely to be scar. When generating consensus segmentations, such disagreement problems are solved by establishing some common ground.

The second reference standard of obtaining locations of enhanced regions with fixed models, *n*-SD and FWHM methods, was performed by fixed thresholding on the atrial wall. The wall was approximated by dilating the endocardial LA segmentation by three pixels. Both the SD and FWHM require a region of normal myocardium and results can vary with a different selection. The region within normal myocardium was thus carefully selected to exclude any enhanced pixels. The FWHM was implemented as described in [12] with manual selection of an enhanced region and 50% of the maximum intensity in this region used as a threshold. In some rare instances the 50% cut-off was re-adjusted. Note region-growing was used to obtain the final segmentation result and this ensured pixel connectivity and coherence in the result.

A range of different metrics for measuring algorithm performance were explored. The Dice metric was selected for measuring volumetric overlap. It was computed regionally on carefully selected enhanced areas where the consensus segmentation was in agreement for scar or not scar (i.e. artefact). A surface metric was also selected for measuring the amount of overlap in segmentations. All segmentations were projected onto their LA surfaces and the cumulative Euclidean distance between the corresponding scar labels on the surface was represented as RMSE error. Furthermore, a third measure looked at computing the difference of fibrosis/scar volumes in segmentations. This assessed the quantifiable infarct reported by each method.

Segmentation of scar from LGE CMR images poses various challenges and thus an overlap assessment is not alone sufficient. To detect which false positives and negatives are more prevalent, regional assessments of aortic wall and navigator beam artefacts were provided. Regions containing these artefacts were carefully chosen and an overlap assessment was made for each method. This highlights how algorithms fare with regularly enhancing features of LGE CMR images. Further, the framework provided a grading for each post-ablation image in its database. Algorithms can select images of a specific quality when using the framework through the web-based interface.

A limitation of the framework is the size of the image database. It is sufficient for most purposes, for instance assessing an algorithm initially against different protocols and acquisition parameters. The website hosting the image database is scalable and can easily be scaled to include additional images when they become available. A second limitation is the performance metrics. Dice is known to be highly sensitive to mismatch of small structures and thus can disproportionately penalise algorithms in some instances. The surface based metric (i.e. RMSE) also has an important limitation; images with a large amount of false-positive scar detected yield a very low RMSE error. This is because there are false positive points in the vicinity of most surface points labelled by raters as scar making the distance error small. However, this limitation can be overcome if the surface measure is combined and read with the volume difference measure. This gives a truer picture of the segmentation.

### Evaluated algorithms

Some methods make assumptions about the intensity distribution of enhanced pixels within atrial myocardium. Modelling the distribution with a statistical distribution such as a Gaussian is a common technique. Prior to modelling, some normalise atrial myocardium intensities to the easily observable atrial blood pool by taking its average (see Eq. 1). Table 6 summarises the approaches undertaken by each method. To compare the proposed models with the true intensity distribution of scar, the distribution of intensities in the consensus segmentation was investigated in Section 'Image quality on segmentation’ and shown in Figure 9. A limitation with the Gaussian approach is that the Gaussian function diminishes at its tail with increasing enhancement; greater enhancement is more likely to be scar. The sigmoid curve has an open-end and can overcome this limitation. Normalisation can be important as intensities in CMR do not correspond to tissue types as they do in computed tomography (CT) imaging. However, modelling enhancement and normalising it is not alone sufficient given the dynamic range and using other modes of information might be necessary. Examples within the evaluated methods include extracting contextual information from a pixel neighbourhood (KCL, SY), exploiting pixel connectivity (IC), adjusting the fixed model for every slice (YL, UTA), utilising a feature space (SY, UTB).

**Table 6:** Enhancement normalisation models adopted (if any) in each method

**Method**	**Normalisation**	**Model**
IC	Y	Sigmoid
MV	Y	Gaussian
SY	N	Gaussian
HB	N	Gaussian
YL	N	None
KCL	Y	Gaussian
UTA	N	Gaussian
UTB	Y	Gaussian

Y = normalisation to blood pool intensity, N = no normalisation.

All the methods outperformed the FWHM and *n*-SD methods in our evaluation. There was also significant improvement offered in some: pre-ablation (YL vs. 4-SD, paired *t*-test: *p* < 0.05) and post-ablation (KCL vs. 2, 4, 6-SD, paired *t*-test: *p* < 0.05). This suggests that a fixed model for scar is not a viable solution and improvements can be made. There is further evidence for this as evaluated methods YL and UTA using simple thresholding find it necessary to adjust thresholds for each slice and achieve significant improvements over fixed models (paired *t*-test *p* < 0.05).

Segmentation of LA myocardial wall is an important step before segmenting scar. The LA wall is much more thin and flexible than that of the ventricle. It is known to be 2.5 mm in thickness [35]. Also in areas of no contrast the LA wall is impossible to visualise and thus can only be approximated. In the evaluated algorithms, there were several that used a fixed distance from the endocardial LA border (IC, MV, SY, HB, KCL) of which two (IC, HB) computed this distance directly using an Euclidean distance measure and the rest (MV, SY, KCL) used morphological dilation. However, there were three methods (YL, UTA, UTB) that used a manual delineation of the wall. From the artefact analysis of Figure 8 it is also YL, UTA and UTB that have the least amount of aortic wall and navigator artefacts. The aortic wall problem is very minimal in YL, UTA and UTB, whilst there is yet some navigator beam artefact. This is suggestive of the fact that a good LA wall segmentation can counteract to a great extent the aortic wall problem but also overall improves LGE CMR segmentation.

Pre-ablation enhancement that is likely to be due to fibrosis is more challenging to detect than post-ablation enhancement due to scar. One reason is fibrosis appears more diffuse with greater overlap with normal myocardium. Algorithms IC, YL, UTA and UTB only show reasonable overlap (Dice, RMSE and |*δV*|), with YL’s results available on a smaller cohort (10 out of 30) and both YL and UTA requiring significantly longer processing times than the rest. Fixed models (4-SD an FWHM) fare poorly in comparison. This comes as no surprise as with greater overlap of intensities for normal myocardium and fibrosis in pre-ablation, a fixed model is bound to fail. Even with an optimal separation between the distributions computed, further processing is needed and this is included in IC (pixel connectivity) and SY (contextual information) algorithms. Others have similar processing steps but were developed primarily for post-ablation enhancement and thus has a bias (MV and KCL). In post-ablation enhancement, most evaluated algorithms demonstrated that good segmentation is possible. This is true in the case of automated (IC, SY, MV, HB, KCL, UTB) and semi-automated ones (YL, UTA). Fixed models had lesser accuracy with a difference of at least 10 points on the Dice compared with some methods (MV, KCL, YL), but their performance was better compared to performance in pre-ablation.

### Future algorithms

The aim of this work is to provide a standardised methodology and framework for evaluating state-of-the-art algorithms that was made available to the wider community through a web-based interface. The framework has potential that upcoming state-of-the-art algorithms can utilise it to evaluate their performance. That would enable algorithms to be benchmarked against other algorithms. Eight different algorithms were evaluated with the proposed framework, three of which are published or slightly modified versions of published techniques ( [5,15,18]). This gives the framework some standing and acceptability and gives future algorithms a sensible ground for testing. Also to our knowledge, this is the first proposed framework of its kind for testing LGE CMR algorithms.

## Conclusions

CMR continues to play an increasingly important role for quantifying LA fibrosis and scar before and after an ablation procedures for AF. LGE CMR is a challenging imaging technique with variation often seen in image and enhancement quality. Currently, algorithms have only been tested on centre- and vendor-specific images. Their suitability and performance in images from other centres or vendors is not very clear. Also, algorithms cannot be tested on the same datasets and thus they cannot be cross-compared. The proposed framework evaluated 8 different algorithms and measured their performance on a common scale. Reference standards for evaluation were established. Following evaluation, no algorithm was deemed clearly better than the others. This leaves scope to push for further algorithmic developments in LA fibrosis and scar imaging. Benchmarking of future scar segmentation algorithms is important. The proposed framework remains publicly available for accessing the image database, uploading algorithm segmentations for evaluation and contributing manual segmentations for improving the reference standard.

## Competing interests

The authors declare that they have no competing interests.

## Authors’ contributions

RK evaluated the algorithms, analysed the results and drafted the manuscript. RJH and MB performed graphical analysis and evaluation. JC and DP performed analysis of the results, authored two algorithms and provided their outputs. ZC, AU, YA, EP, PA, SO performed fibrosis and scar evaluation. AH, YL, WB, WS, YG, HP, PR, AT, DR authored the algorithms and provided their outputs. DPT, RM and KR organized the challenge and provided expert advice together with RR and TS. KR also provided supervision and revised the manuscript critically for important intellectual content. All authors read and approved the final manuscript.
